# Application of Peroxide of Hydrogen for Medicinal Purposes

**Published:** 1882-10

**Authors:** 


### Application of Peroxide of Hydrogen for Medicinal Purposes.

The peroxide of hydrogen has not hitherto played a conspicuous part in therapeutics. The reason for that may be, that formerly pure and durable solutions were not to be had at a reasonable figure. Price, however, is no longer an impedient to its use, and the tendency of the peroxide of hydrogen, as at present obtainable, to decompose, can be considerably restricted; possibly peroxide of hydrogen turned into simple water may formerly have led to wrong conclusions. Peroxide of hydrogen, if preserved in the dark, and in a temperature not exceding 25 degrees C. (77 degrees F.), keeps unaltered for months. For ascertaining its titer of active oxygen, a normal solution of permanganate of potash is requisite; it would be advisable to fix a minimum titer of active hydrogen. It is to be supposed that peroxide of hydrogen, like chloride, bromide, and permanganate of potash, is poison to the smallest organisms (bacteria); exact comparative experiments with a view to ascertain this are much to be desired, considering the importance of the matter. Experiments with yeast, instituted by the lecturer, had very favorable results, and proved that the germs of the yeast are entirely killed by peroxide of hydrogen, even when greatly diluted.

As regards the fitness of peroxide of hydrogen for treating wounds, caused by syphilitic, scrofulous, and tuberculous ulcers, favorable experience has been gleaned by a physician at Hanover. It is probable that peroxide of hydrogen will do good service in the shape of spray in making operations and ligatures; this would be important, considering the affect which carbolic acid spray often has on operators and patients.

The great advantages possessed by peroxide of hydrogen, as compared with other media of disinfection, are:

1. Complete absence of smell.

2d. Yielding oxygen without leaving any other residuum but pure water.

3d. Absence of injurious influence on the organism.

The workmen occupied in making the peroxide of hydrogen get exceedinginly delicate hands, and wounds heal visibly under its influence.

There further seems room for employing the peroxide of hydrogen as a means of disinfecting sick chambers and generally for purifying the air. It would be advisable to spread by means of a rafraichisseur spray of diluted peroxide of hydrogen by way of trial.

Attention must also be drawn to the use of peroxide of hydrogen in dentistry, as has in the first place been done by C. Sauer (*Quarterly Review of Dentistry*, 1879, No. iv.). Sauer made use of the peroxide of hydrogen with success in bleaching discolored and carious teeth. In cases where the teeth are covered with colored matter (*Lichen dentalis*, etc.), he employs peroxide of hydrogen in conjunction with finely levigated pumice stone, as a means of cleaning in place of water. Teeth, the native channels of which were filled with colored matter, became somewhat paler after several applications. A suitable liquid for cleaning teeth and mouth is prepared by mixing 1 part of 3 per cent. peroxide of hydrogen with 10 parts of water. In case of carious teeth, the peroxide of hydrogen on wadding was locally used with advantage.

